# Risk of chronic kidney disease in patients with a hyperglycemic crisis as the initial presentation of type 2 diabetes

**DOI:** 10.1038/s41598-024-67678-3

**Published:** 2024-07-20

**Authors:** Chun-Ta Huang, Chih-Hsin Muo, Fung-Chang Sung, Pei-Chun Chen

**Affiliations:** 1 Division of Endocrinology and Metabolism, Department of Internal Medicine, Mackay Memorial Hospital, Taipei City, 104217 Taiwan; 2 Department of Medicine, Mackay Medical College, New Taipei City, 252005 Taiwan; 3 Management Office for Health Data, China Medical University Hospital, Taichung City, 404328 Taiwan; 4 Department of Health Services Administration, China Medical University College of Public Health, 100 Jingmao Road Section 1, Beitun Dist., Taichung, 406040 Taiwan; 5 Department of Food Nutrition and Health Biotechnology, Asia University, Taichung, 413305 Taiwan; 6 International Master Program for Public Health, China Medical University, Taichung, 406040 Taiwan

**Keywords:** Chronic kidney disease, Diabetic ketoacidosis, Diabetic kidney disease, Hyperglycemic crisis, Hyperglycemic hyperosmolar state, Type 2 diabetes, Chronic kidney disease, Outcomes research

## Abstract

Limited data exist on long-term renal outcomes in patients with hyperglycemic crisis (HC) as initial type 2 diabetes presentation. We evaluated the risk of chronic kidney disease (CKD) development in those with concurrent HC at diagnosis. Utilizing Taiwan’s insurance claims from adults newly diagnosed with type 2 diabetes during 2006–2015, we created HC and matched non-HC cohorts. We assessed incident CKD/diabetic kidney disease (DKD) by 2018’s end, calculating the hazard ratio (HR) with the Cox model**.** Each cohort comprised 13,242 patients. The combined CKD and DKD incidence was two-fold higher in the HC cohort than in the non-HC cohort (56.47 versus 28.49 per 1000 person-years) with an adjusted HR (aHR) of 2.00 (95% confidence interval [CI] 1.91–2.10]). Risk increased from diabetic ketoacidosis (DKA) (aHR:1.69 [95% CI 1.59–1.79]) to hyperglycemic hyperosmolar state (HHS) (aHR:2.47 [95% CI 2.33–2.63]) and further to combined DKA-HHS (aHR:2.60 [95% CI 2.29–2.95]). Subgroup analysis in individuals aged ≥ 40 years revealed a similar trend with slightly reduced incidences and HRs. Patients with HC as their initial type 2 diabetes presentation face a higher CKD risk than do those without HC. Enhanced medical attention and customized interventions are crucial to reduce this risk.

## Introduction

Diabetic ketoacidosis (DKA) and hyperglycemic hyperosmolar state (HHS) represent life-threatening hyperglycemic crises (HC) in patients with diabetes^1^. Although commonly seen in patients with preexisting diabetes, up to 20% of cases occur in those newly diagnosed^2–4^. Individuals with HC episodes face higher risks of subsequent morbidity and mortality compared with those without such episodes^5–7^. However, limited research explores the risk of chronic complications in patients experiencing HC during diabetes diagnosis, underscoring the need for further investigation.

Previous studies have reported an increased risk of subsequent stroke^8,9^, cardiovascular events^10^, long-term mortality^11,12^, and end-stage renal disease^13^ in patients with diabetes who have experienced an HC. Nevertheless, these studies overlook the confounding effect of glycemic control on the development of diabetes-related chronic complications. Moreover, type 1 and type 2 diabetes possess distinct pathophysiologies and should not be considered as a single entity; however, only two studies exclusively included patients with type 2 diabetes^8,9^, and none were conducted in patients with newly diagnosed diabetes. In contrast, an Italian multicenter cohort study demonstrated that patients with DKA upon type 1 diabetes onset were not at an increased risk of diabetic retinopathy or albuminuria^14^. The reasons behind these conflicting findings and the impact of HC that occurs in patients newly diagnosed with type 2 diabetes remain to be elucidated.

Therefore, in this study, we aimed to examine the risk of developing chronic kidney disease (CKD), one of the major chronic complications of diabetes, in patients experiencing HC upon type 2 diabetes diagnosis. We hypothesized that HC occurring upon type 2 diabetes diagnosis is associated with a higher risk of developing CKD. Our findings could hold significance if this unique initial presentation of type 2 diabetes can be utilized to identify patients at increased risk of developing CKD; moreover, it could facilitate the implementation of preventive interventions to reduce CKD risk in this population.

## Methods

### Data sources

In this study, we utilized Taiwan’s insurance claims data to identify adults newly diagnosed with type 2 diabetes between 2006 and 2015. We used the Taiwan National Health Insurance Research Database (NHIRD), a comprehensive repository established in 1995 that covers over 99% of the nation’s residents^15,16^. The NHIRD comprises encrypted records encompassing sociodemographic information, household income, residency, outpatient/inpatient care, and prescribed medications. Diseases in the NHIRD are coded using the International Classification of Diseases, Ninth Revision, Clinical Modification (ICD-9-CM) before 2016 and Tenth Revision (ICD-10-CM). Previous investigations have validated the NHIRD’s accuracy and reliability for population-based studies^17,18^.

### Design setting and study cohorts

**Figure 1:**
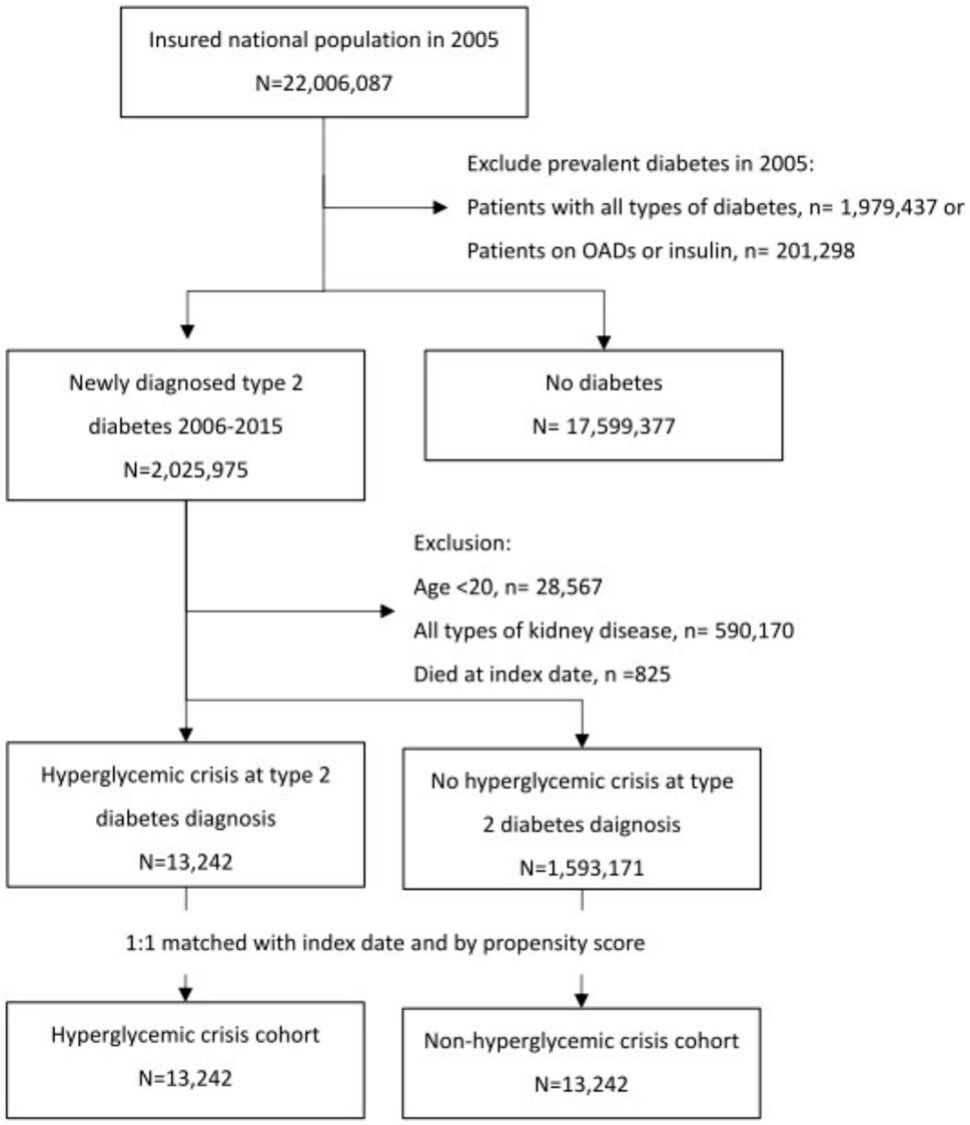
Flow chart showing the establishment of the hyperglycemic crisis cohort and propensity score matched non-hyperglycemic crisis cohort from the National Health Insurance Research Database, Taiwan, 2006–2018. OAD, oral anti-diabetic agent.

From the NHIRD, we used the ICD-9-CM or ICD-10-CM and Anatomical Therapeutic Chemical Codes (Tables S1 and Table S2) to identify newly diagnosed cases with type 2 diabetes (ICD-9-CM codes: 250. X0 or 250. × 2) during the study period (Fig. 1). We excluded individuals who were diagnosed with diabetes or using glucose-lowering drugs before the study period, younger than 20 years of age, had a history of kidney disease including benign or malignant neoplasms of the kidney, chronic kidney disease of any cause, glomerulonephritis, nephrotic syndrome, urolithiasis, and congenital renal anomalies, or were deceased at baseline. Individuals with concurrent HC at the time of their type 2 diabetes diagnosis (first-time type 2 diabetes diagnosis appeared with at least one of the following ICD-9-CM codes: 250.10, 250.12, 250.20, or 250.22) were categorized into the HC cohort. The clinical diagnostic criteria for HC were based on previous guidelines^19,20^, and our data were based on diagnostic codes from all medical facilities regardless of inpatient/outpatient care or emergency department visits. Regarding individuals without HC, we selected a control (non-HC) cohort with the same sample size frequency matched by diagnosis year and propensity score. The propensity scores were calculated using multivariable logistic regression for each person at baseline, including sex, age, type of residence, enrollment category, monthly income, comorbidities, and use of angiotensin-converting enzyme inhibitors *(*ACEis) or angiotensin II receptor blockers (ARBs)*.* Comorbidities were diseases with at least two outpatient claims or one inpatient claim within 1 year of the index date and included hypertension, heart failure, coronary artery disease, ischemic stroke, transient ischemic attack, peripheral arterial disease, hyperlipidemia, obesity, and malignancy. Variables with a between-group standardized mean difference of < 0.1 were considered well-balanced^21^. Sodium-glucose cotransporter-2 inhibitors were not included due to their unavailability in Taiwan during the enrollment period.

The two cohorts were followed up until the end of 2018. Our outcome of interest was the combined incidence of CKD (ICD-9-CM codes: 585–586; ICD-10-CM code: N184–N186, N189–N19) or diabetic kidney disease (DKD) (ICD-9-CM codes: 250.40 or 250.42; ICD-10-CM: E112), or both. In Taiwan, it is generally accepted that CKD is characterized by a decreased glomerular filtration rate of less than 60 mL/min per 1.73 m^2^ and/or markers of kidney damage that persist for longer than 3 months. DKD is a clinical diagnosis that refers to those cases with CKD presumed to be caused by diabetes. Clinicians are free to use whichever code is appropriate based on their judgment. Hence, the data of patients with diabetes and CKD may be coded using CKD, DKD, or both. As DKD is within the spectrum of CKD and our aim was to explore the risk of CKD, we used both codes to maximize the probability of capturing our main outcome of interest.

### Institutional review board statement

This study was approved by the Institutional Review Board of Mackay Memorial Hospital (approval number: 22MMHIS382e). Our study was performed in accordance with the Declaration of Helsinki and all our methods were carried out under relevant guidelines and regulations. As all personal identifications in the database were encrypted and unidentifiable, the requirement for informed consent from the insured individuals was waived.

### Statistical analysis

Baseline characteristics and comorbidities were compared between the HC and non-HC cohorts using Pearson’s χ^2^ test for categorical variables and Student’s *t*-test for continuous variables. The HC cohort comprised three sub-cohorts: patients with DKA (ICD-9-CM codes: 250.10 or 250.12), HHS (ICD-9-CM codes: 250.20 or 250.22), and combined DKA and HHS (DKA-HHS). The cumulative incidence rate of combined CKD and DKD between the HC and non-HC cohorts, as well as between the HC sub-cohorts, was estimated and plotted using the Kaplan–Meier method. Inter-group differences were examined using the log-rank test. Cox proportional hazards regression analysis was used to calculate the crude hazard ratio comparing the HC and non-HC cohorts; moreover, the adjusted hazard ratio (aHR), along with its corresponding 95% confidence interval (CI), was used for the combined occurrence of CKD and DKD. The aHR was estimated after adjusting for age, sex, socioeconomic factors, and significant comorbidities. As patients who died before the event occurred will never be coded with CKD and/or DKD, the competing risk of death was managed using the Fine-Grey analysis model to estimate the sub-distribution hazard ratio (sHR)^22^. To address potential misclassification and pollution bias from claims data, where type 1 diabetes might have been incorrectly recorded as type 2 diabetes, we performed a subgroup analysis to estimate the combined incidence rates of CKD and DKD for individuals aged 40 years and older. We selected this cutoff because the incidence of type 1 diabetes drops significantly after age 40 years and remains relatively low in this age group. This approach helps to minimize the risk of pollution bias in our results^23^. To validate that the incident events were not the result of undiagnosed preexisting CKD or DKD, a supplementary sensitivity analysis was performed by excluding outcomes that occurred within 6 months after the diagnosis of type 2 diabetes. Finally, we conducted a nested case–control analysis to explore the risk factors for CKD or DKD, including DKA, HHS, acute kidney injury, nonsteroidal anti-inflammatory drug use, and ACEi or ARB use. Medication use was stratified based on the prescription length into three categories: no exposure (0 days), 90 days or less, and more than 90 days. Statistical analyses were performed using the Statistical Package for SAS V. 9.4 (SAS Institute, Cary, North Carolina, USA), and a two-sided *P*-value of less than 0.05 was considered statistically significant.

### Ethics-approval and consent to participate

This study was approved by the Institutional Review Board of Mackay Memorial Hospital (approval number: 22MMHIS382e) and informed consent was waived.

### Presentation at a meeting

The current study was presented as an e-poster at the IDF 2022 Congress in Lisbon, Portugal, on December 5–8.

## Results

### Cohort characteristics

There were 13,242 participants in each cohort (Fig. 1), and their baseline characteristics are shown in Table 1. The mean age of the study participants was approximately 54 years (men: 62%). Compared with the non-HC cohort, the HC cohort had a lower income and higher prevalence of malignancy (6.24% vs. 2.99%, respectively); however, they had a lower prevalence of hypertension (32.7% vs. 37.5%, respectively) and hyperlipidemia (13.2% vs. 20.7%, respectively). The two cohorts did not differ in the proportion of patients who received either ACEis or ARBs.

**Table 1:** Baseline characteristics of participants with and without hyperglycemic crisis at diabetes diagnosis from the National Health Insurance Research Database, Taiwan, 2006–2018.

Variable	Hyperglycemic crisis					
	Yes N = 13,242		No N = 13,242		*P*-value	SMD
	n	%	n	%		
Sex					0.0006	
Men	8217	62.1	8487	64.1		0.042
Women	5025	37.9	4755	35.9		0.042
Age, years					0.004	
< 40	2976	22.5	2823	21.3		0.028
40–54	4210	31.8	4438	33.5		0.037
55–64	2298	17.4	2357	17.8		0.012
> 65	3758	28.4	3624	27.4		0.023
Mean (SD)	54.2	(17.6)	54.1	(16.7)	0.566	0.007
Urbanization level					0.322	
1 (highest)	7511	56.7	7627	57.6		0.018
2	4256	32.1	4190	31.6		0.011
3 (lowest)	1475	11.1	1425	10.8		0.012
Occupation					< 0.0001	
Home	3139	23.7	2783	21.0		0.065
Blue collar	3447	26.0	4333	32.7		0.147
White collar	3824	28.9	4024	30.4		0.033
Other	2832	21.4	2102	15.9		0.142
Income^a^					< 0.0001	
≤ 20,000	8594	64.9	7673	57.9		0.143
20,001–39,999	2861	21.6	3181	24.0		0.058
≥ 40,000	1787	13.5	2388	18.0		0.125
Comorbidity						
HTN	4325	32.7	4959	37.5	< 0.0001	0.100
Heart failure	762	5.75	580	4.38	< 0.0001	0.063
CAD	1433	10.8	1706	12.9	< 0.0001	0.064
Ischemic stroke/TIA	1262	9.53	1027	7.76	< 0.0001	0.063
PAD	290	2.19	330	2.49	0.104	0.020
Hyperlipidemia	1742	13.2	2742	20.7	< 0.0001	0.202
Obesity	92	0.69	136	1.03	0.003	0.036
Malignancy	826	6.24	396	2.99	< 0.0001	0.155
Treatment						
ACEi	457	3.45	515	3.89	0.058	0.025
ARB	914	6.90	1059	8.00	0.0007	0.042

ACEi, angiotensin-converting enzyme inhibitor; ARB, Angiotensin receptor blocker; CAD, coronary artery disease; HTN, hypertension; PAD, peripheral arterial disease; SD, standard deviation; SMD, standard mean difference; TIA, transient ischemic attack.

^a^Monthly income in New Taiwan dollars (NTD): 20,000 NTD is equivalent to US$650.

### Combined incidence of CKD and DKD

**Figure 2:**
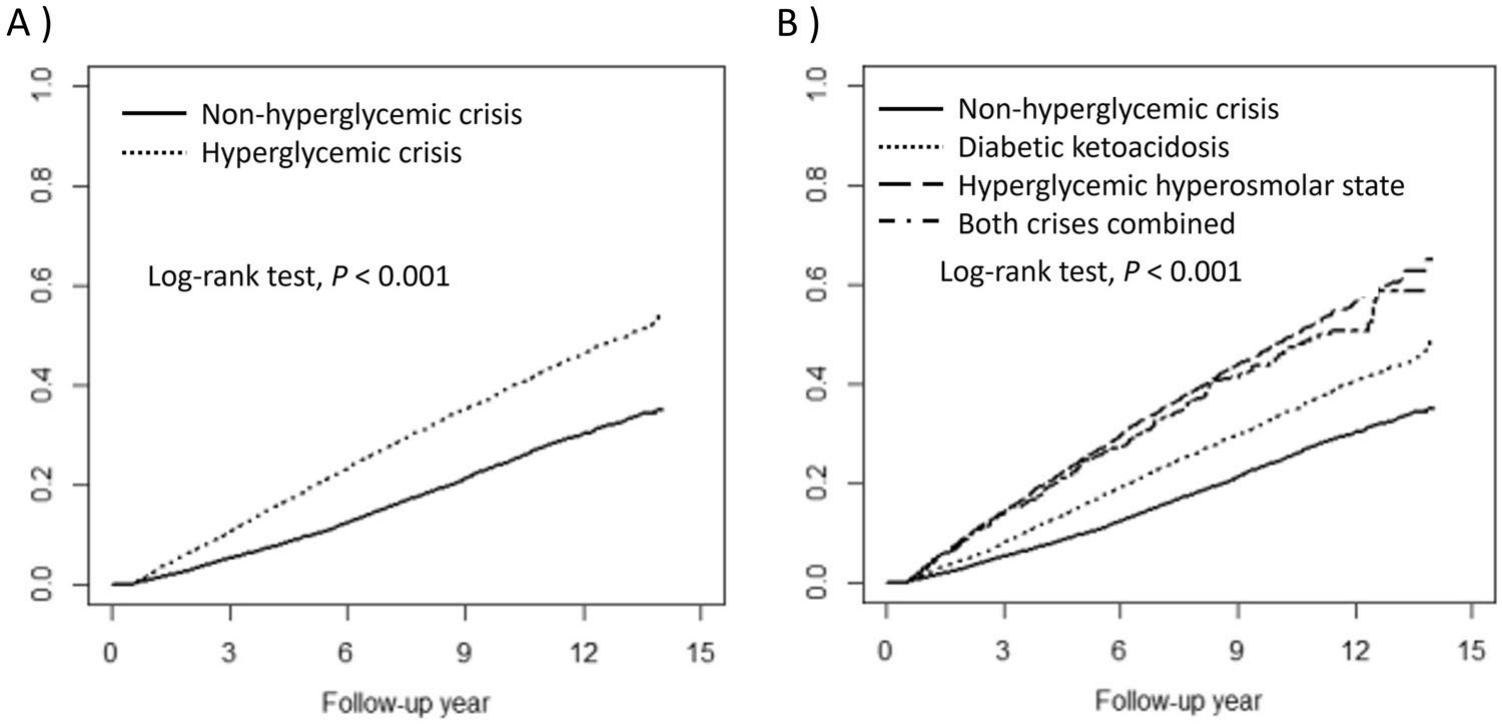
Group comparisons of the Kaplan–Meier estimated cumulative incidence of combined chronic kidney disease and diabetic kidney disease between hyperglycemic crisis (HC) and non-hyperglycemic crisis (non-HC) cohorts (**A**), and among hyperglycemic hyperosmolar state (HHS), diabetic ketoacidosis (DKA), combined DKA-HHS sub-cohorts and non-HC cohorts (**B**), from the National Health Insurance Research Database, Taiwan, 2006–2018.

Table 2 depicts the combined incidence of CKD and DKD in the HC cohort, its sub-cohorts, and in the non-HC cohort. The HC cohort comprised mainly patients with DKA (55.1%) and HHS (39.2%), with a few cases of combined DKA-HHS (5.7%). There were 4106 (31%) and 2735 (20.7%) events observed in the HC and non-HC cohorts during median follow-ups of 4.97 years and 7.15 years, respectively. This corresponded to incidence rates of 56.47 and 28.49 per 1000 person-years, respectively. The cumulative incidence of CKD and DKD was significantly greater in the HC cohort than in the non-HC cohort (Fig. 2A). Within the HC cohort, the cumulative incidence was higher in patients with HHS and combined DKA-HHS than in those with DKA (Fig. 2B). The aHR among the HC sub-cohorts increased from 1.69 (95% CI 1.59–1.79) for DKA to 2.47 (95% CI 2.33–2.63) for HHS and 2.60 (95% CI 2.29–2.95) for combined DKA-HHS. In the sub-distribution hazard models, the sHRs were attenuated but remained significantly higher in the main HC cohort and its sub-cohorts.

**Table 2:** Combined incidence of chronic kidney disease and diabetic kidney disease and related hazard ratios with 95% confidence intervals in participants with and without hyperglycemic crisis at diabetes diagnosis from the National Health Insurance Research Database, Taiwan, 2006–2018.

Outcome	Hyperglycemic crisis									
	No N = 13,242		All N = 13,242		DKA N = 7297		HHS N = 5185		DKA-HHS N = 760	
	n	Rate^a^	N	Rate^a^	n	Rate^a^	n	Rate^a^	n	Rate^a^
CKD or DKD	2735	28.49	4106	56.47	2101	46.33	1731	73.62	274	71.19
Crude HR	Ref		1.98	(1.89–2.08)	1.63	(1.54–1.72)	2.60	(2.45–2.76)	2.53	(2.23–2.87)
Adjusted HR^b^	Ref		2.00	(1.91–2.10)	1.69	(1.59–1.79)	2.47	(2.33–2.63)	2.60	(2.29–2.95)
Crude SHR	Ref		1.65	(1.57–1.73)	1.49	(1.41–1.57)	1.82	(1.72–1.94)	2.11	(1.86–2.39)
Adjusted SHR^b^	Ref		1.68	(1.60–1.76)	1.54	(1.46–1.63)	1.81	(1.70–1.93)	2.16	(1.91–2.46)

All *P*-values are highly statistically significant and < 0.001.

CKD, chronic kidney disease; DKA, diabetic ketoacidosis; DKD, diabetic kidney disease; HHS, hyperglycemic hyperosmolar state; HR, hazard ratio; SHR, sub-distribution hazard ratio.

^a^Per 1000 person-years.

^b^Adjusted for age, sex, socioeconomic factors, and significant comorbidities at baseline.

### Subgroup analysis in individuals aged 40 years and older

There were 10,266 (77.5%) and 10,419 (78.7%) individuals aged 40 years and older in the HC and non-HC cohorts, respectively (Table 3). This subgroup comprised 5104 (49.7%) individuals with DKA and 4591 (44.7%) with HHS in the HC cohort. A similar increase in the HR between the HC and non-HC cohorts, compatible with the result in our primary analysis, was observed in this age group. The aHR among the HC sub-cohorts also showed a stepwise escalation from 1.62 (95% CI 1.51–1.72) for DKA to 2.33 (95% CI 2.19–2.49) for HHS and 2.59 (95% CI 2.25–2.98) for combined DKA-HHS.

**Table 3:** Combined incidence of chronic kidney disease and diabetic kidney disease and related hazard ratios with 95% confidence intervals in participants aged 40 years and older with and without hyperglycemic crisis at diabetes diagnosis from the National Health Insurance Research Database, Taiwan, 2006–2018.

Outcome	Hyperglycemic crisis									
	No N = 10,419		All N = 10,266		DKA N = 5104		HHS N = 4591		DKA-HHS N = 571	
	n	Rate^a^	N	Rate^a^	n	Rate^a^	n	Rate^a^	n	Rate^a^
CKD or DKD	2375	32.48	3307	63.31	1535	51.69	1546	78.10	226	82.21
Crude HR	Ref		1.96	(1.86–2.06)	1.60	(1.50–1.70)	2.43	(2.28–2.59)	2.57	(2.23–2.95)
Adjusted HR^b^	Ref		1.94	(1.84–2.05)	1.62	(1.51–1.72)	2.33	(2.19–2.49)	2.59	(2.25–2.98)
Crude SHR	Ref		1.55	(1.47–1.63)	1.41	(1.32–1.50)	1.65	(1.55–1.76)	2.08	(1.81–2.39)
Adjusted SHR^b^	Ref		1.57	(1.49–1.66)	1.44	(1.35–1.54)	1.66	(1.56–1.77)	2.12	(1.85–2.44)

All *P*-values are highly statistically significant and < 0.001.

CKD, chronic kidney disease; DKA, diabetic ketoacidosis; DKD, diabetic kidney disease; HHS, hyperglycemic hyperosmolar state; HR, hazard ratio; SHR, sub-distribution hazard ratio.

^a^Per 1000 person-years.

^b^Adjusted for age, sex, socioeconomic factors, and significant comorbidities at baseline.

### Sensitivity analysis

The sensitivity analysis demonstrated that the patterns of CKD or DKD development 6 months after the diagnosis of type 2 diabetes were consistent with the findings of the primary analysis (Table S3).

### Nested case–control analyses

The nested case–control analysis showed that the risk of developing CKD or DKD was significantly higher for patients with a history of hyperlipidemia (adjusted odds ratio [aOR] 1.22; 95% CI 1.15–1.30), acute kidney injury (aOR 1.33; 95% CI 1.18–1.50), DKA (aOR 1.56; 95% CI 1.47–1.66), and HHS (aOR 1.75; 95% CI 1.64–1.86). Compared with patients who did not receive ACEis or ARBs, those who had received treatment with ACEis or ARBs also had a higher risk, with an aOR of 1.93 (95% CI 1.75–2.13) for those treated for 90 days or less and an aOR of 1.69 (95% CI 1.57–1.82) for those who were treated for more than 90 days (Table S4).

## Discussion

This population-based cohort study revealed a higher risk of incident CKD and/or DKD in patients with HC as their initial presentation of type 2 diabetes than in patients who present type 2 diabetes without HC. This association remained consistent across all HC sub-cohorts and stayed significant in the subgroup analysis for those aged 40 years and older. The risk was higher in patients with HHS and in those with both DKA and HHS than in those with DKA. This association remained robust after excluding cases that appeared within 6 months of diabetes diagnosis. Our nested case–control analysis corroborates that, compared with patients with type 2 diabetes who did not develop CKD or DKD, those who did were more likely to have experienced HC upon diabetes diagnosis.

The surprisingly high proportion of patients having DKA instead of HHS on the initial presentation of their type 2 diabetes in our cohort may raise concerns of pollution bias by the presence of patients with type 1 diabetes. Although there is currently no data reporting the proportion of DKA versus HHS in patients with HC as the initial presentation of type 2 diabetes, we believe that our findings are valid because previous studies have also reported a high percentage of newly diagnosed type 2 diabetes among patients with DKA. In an early study of 141 episodes of DKA in a tertiary referral center in Taiwan, 32 (22.7%) episodes were caused by newly diagnosed diabetes^24^. Twenty-five of the newly diagnosed patients were followed for at least 12 months, and 11 (44%) of them were not using insulin and exhibited metabolic features of type 2 diabetes. A recent study that retrospectively reviewed the medical records of consecutive patients with index DKA in four general hospitals in Qatar showed that 442 (48%) of them had type 2 diabetes^25^. Of the 324 patients with DKA and newly diagnosed diabetes, 176 (54.3%) had type 2 diabetes, and 93 (52.8%) were Asian. We speculate that the excessive DKA cases observed in our study and previous studies may be attributed to ‘ketosis-prone diabetes (KPD)’^26^. This syndrome is characterized by the acute onset of severe hyperglycemia with ketoacidosis, necessitating hospital admission and treatment. However, it often undergoes spontaneous remission, with patients maintaining long-term insulin independence several weeks after discharge^27^. Initially identified in individuals of African descent and African Americans^28^, KPD is now recognized as a significant clinical entity in Asian populations^27^. Patients with KPD are typically young or middle-aged and predominantly male^29^, consistent with the clinical characteristics of our study population. Another reason we believe that we secured the cases of type 2 diabetes in our study is due to the unique characteristics of the NHIRD. In Taiwan, type 1 diabetes is classified as a catastrophic illness by the National Health Insurance Administration. When a physician diagnoses a patient with such a condition, the patient can apply for a catastrophic illness certificate by submitting the necessary documentation. Upon issuance, this certification is recorded on the patient’s National Health Insurance Card. During the validity period of this certificate, patients are exempt from co-payment of outpatient or inpatient care related to the certified illness^30^. However, this exemption is contingent upon the physician using the correct ICD codes, as is the case with type 1 diabetes. An incorrect ICD coding that misclassifies type 1 diabetes as type 2 diabetes would prevent patients with type 1 diabetes from receiving exemptions for medical expenses, a scenario which is unlikely to occur in real-world practice or in the NHIRD. Moreover, the result of our subgroup analysis for those aged 40 years and older, which comprised nearly 78% of all patients, did not differ from our main findings. As the incidence of type 2 diabetes increases with age in Taiwan, and the incidence of type 1 diabetes over 40 years of age is remarkably low (0.02 per 100,000 population)^23^, the possibility that our findings are biased due to the presence of patients with type 1 diabetes is negligibly low.

Our findings align with those of previous Taiwan NHIRD studies demonstrating the detrimental effects of HC in patients with diabetes^8–13^. The present research adds value to the previous literature in several aspects. First, unlike previous studies that examined diabetes as a single entity^10–13^, we focused exclusively on type 2 diabetes and examined the individual effects of different types of HC. Given the distinct pathophysiologies underlying DKA and HHS, as well as those underlying type 1 and type 2 diabetes, our study design may ensure a more robust association between HC and CKD. The higher risk of CKD and/or DKD observed in patients with HHS or those with combined DKA-HHS than in patients with isolated DKA also aligns with previous studies reporting worse in-hospital outcomes in patients with combined DKA-HHS than in those with isolated DKA or HHS^31^. Second, HC typically signifies uncontrolled diabetes^32^, which is a well-recognized risk factor for diabetic complications^33^. Studies not adjusting for patients’ glycemic control may confound the impact of HC on long-term outcomes. We minimized such confounding by focusing on patients newly diagnosed with diabetes, where subsequent glycemic control acts as a mediator or effect modifier that requires no adjustment. Third, this pioneering study investigates the impact of HC on patients newly diagnosed with type 2 diabetes without prior kidney disease, offering potential insights for future clinical practice. Given their increased risk of CKD, patients experiencing HC upon type 2 diabetes diagnosis should receive proactive early preventive measures to mitigate such risk.

Several mechanisms may explain our findings. A longitudinal study on type 2 diabetes in Taiwan highlighted that individuals with lower income levels were more likely to have hospitalization-diagnosed diabetes, although it did not report how many of these patients were diagnosed via HC^34^. Patients with type 2 diabetes who are not diagnosed until hospitalization may be less likely to receive early detection screenings or may lack sufficient awareness of diabetes-related symptoms to seek appropriate healthcare^34^. Although the two study groups in our study did not differ in most baseline characteristics after propensity-score matching, individuals in the HC group exhibited significantly lower income levels than those in the non-HC group. Such difference may suggest that our HC group, compared with the non-HC group, included more individuals facing healthcare inequality, potentially resulting in worse renal outcomes. Furthermore, common risk factors for CKD, such as hypertension and hyperlipidemia, often remain undiagnosed among underprivileged individuals^35^. The paradoxically higher risk of CKD despite a lower prevalence of hypertension and hyperlipidemia in the HC group than in the non-HC group may be a result of more undiagnosed rather than healthier cases in the former group. As our propensity score considered only established comorbidities, the true risk difference of CKD at baseline between the two groups may be unbalanced. This could explain the remaining two-fold higher risk of CKD in the HC group, even after adjusting for income level, comorbidities, and other covariates.

From a biological perspective, our finding may be a consequence of initial priming by hyperglycemia, as the onset of diabetes followed by subsequent insults from acute kidney injury (AKI) during HC ultimately leads to persistent nephron damage. Hyperglycemia-induced epigenetic change can lead to progressive and irreversible renal injury, a phenomenon known as the “metabolic memory of DKD”^36^. Following exposure to hyperglycemia, vascular endothelial cells continue to increase oxidative stress and elicit inflammation even after normalization of blood glucose levels^37^. Owing to the slow progression of type 2 diabetes, the exact duration between disease onset and diagnosis is difficult to ascertain. Previous studies have suggested that the interval between the onset and diagnosis of type 2 diabetes is at least 5 years^38,39^. Moreover, low income is significantly associated with delayed diagnosis and inadequate diabetes care and management^34^. We speculate that patients experiencing HC upon diabetes diagnosis had a longer duration from diabetes onset to diagnosis compared with those who had diabetes diagnosed without HC. Such a latent period aggravates metabolic memory and leads to an increased risk of CKD. Moreover, AKI is a known risk factor for CKD^40–42^ and is common in patients with DKA due to volume depletion^7,43^. Despite this, there is a paucity of data reporting the incidence of AKI during HHS. A higher rate of AKI in patients with HHS is expected as patients with HHS are more likely to be dehydrated than patients with DKA. The occurrence of AKI during HC may exacerbate renal damage and increase the risk of CKD. A recent study revealed a higher incidence of AKI in patients with HHS and combined DKA-HHS than in those with DKA^44^, which may also explain the greater risk of CKD in patients with HHS and combined DKA-HHS than in those with DKA in our study. Nevertheless, we cannot exclude the possibility that our findings result from surveillance bias between the two cohorts. As patients experiencing HC upon diabetes diagnosis have more severe disease compared with those without HC at diagnosis, clinicians should provide more vigilant follow-ups for earlier detection of CKD.

This study had some limitations. First, relying solely on claims data may have led to the misclassification of diseases. However, the accuracy and validity of the NHIRD claims data have been demonstrated previously^15,17,18^, minimizing the impact of misclassification on our results. Second, we could not adjust for covariates unavailable in the NHIRD, including laboratory tests, blood pressure, waist circumference, body mass index, and lifestyle. The potential impact of this missing data remains unassessed. Furthermore, the inherent limitations of the NHIRD also restricted us from determining the severity of DKA and the stage of CKD, which may also have affected our results. Third, in large-sample studies, even minor differences can achieve statistical significance, warranting cautious interpretation. Because we established our study cohorts using a propensity score-matched design and conducted the analysis using stratification, we believe that the observed risk differences between the two cohorts were not a result of overpowering. Finally, the generalizability of our findings may be limited to populations with similar characteristics.

## Conclusions

Patients who experience HC upon type 2 diabetes diagnosis have a higher risk of developing CKD compared with those without HC at diagnosis. As type 2 diabetes and end-stage renal disease are highly prevalent in Taiwan, proactive preventive measures are imperative to mitigate risks in this vulnerable population. These interventions should include early introduction of ACEis or ARBs and sodium-glucose cotransporter-2 inhibitors, stringent control of diabetes and the reduction of other risk factors, and educational programs for continuous diabetes self-care management. Furthermore, healthcare authorities should reinforce government-subsidized diabetes screening programs, especially for the underprivileged, to facilitate early recognition of undiagnosed diabetes and prevent HC incidents.

### Supplementary Information

Supplementary Tables.

## Data Availability

No original data were generated or collected as part of this study. The NHIRD used in this study is available from the Taiwan National Health Insurance Administration, Ministry of Health and Welfare.
