# Supplementary material for: Risk of chronic kidney disease in patients with a hyperglycemic crisis as the initial presentation of type 2 diabetes
Source: Sci Rep. 2024 Jul 20;14:16746. doi: 10.1038/s41598-024-67678-3 (PMC11271453; doi:10.1038/s41598-024-67678-3)
Supplement: Supplementary file 1 — Supplementary Tables. [file 41598_2024_67678_MOESM1_ESM.docx]

**Table S1: Diagnostic codes used for identifying diseases, covariates and outcomes in the study of risk of chronic kidney disease in patients with hyperglycemic crisis as initial presentation of type 2 diabetes from the National Health Insurance Research Database, Taiwan, 2005-2018**

| **Clinical diagnosis** | **ICD-9 CM** | **ICD-10 CM** |
| --- | --- | --- |
| Type 2 diabetes^a^ | 250.x0, 250.x2 | E11 |
| Type 2 diabetes with diabetic ketoacidosis | 250.10, 250.12 | E131 |
| Type 2 diabetes with hyperglycemic hyperosmolar state | 250.20, 250.22 | E110, E130 |
| Hypertension | 401-405 | I10-I15 |
| Heart failure | 402.x1, 404.x1, 404.x3, 428 | I110, I130, I132, I50 |
| Coronary artery disease | 410-414 | I21-I22, I24-I25 |
| Ischemic stroke / Transient ischemic attack | 433-437 | I63, I65-I66, I678, G45-G46 |
| Peripheral artery disease | 440, 443.9, 444.2 | I70, I739, I742-I744, I75 |
| Hyperlipidemia | 272 | E78 |
| Obesity | 278.00, 278.01 | E66 |
| Malignancy | 140-208 | C00-C97 |
| All types of kidney disease^a,b^ | 189, 223.0, 223.1, 236.91, 250.4, 403-404, 580-593, 642.1, 646.2, 753.0-753.3 | NA |
| Chronic kidney disease | 585-586 | N184-N186, N189, N19 |
| Diabetic kidney disease | 250.40, 250.42 | E112 |
| Acute kidney injury | 584.5-584.9 | N170-N172, N178, N179 |

Abbreviations: ICD-9 CM, International Classification of Diseases, Ninth Revision, Clinical Modification; ICD-10 CM, International Classification of Diseases, Tenth Revision, Clinical Modification.

^a^ ICD-9 CM was used to identify study cohorts from claims data of 2005-2015, for inclusion or exclusion during enrollment. Other diagnoses were used for both enrollment and follow-ups as well as for nested case-control analysis. ^b^ Including neoplasm of urinary system, inflammatory disease of urinary system, urolithiasis, diabetic or hypertensive kidney disease, and congenital malformations.

**Table S2: Anatomical Therapeutical Chemical (ATC) codes used for medication ascertainment in the study of risk of chronic kidney disease in patients with hyperglycemic crisis as initial presentation of type 2 diabetes from the National Health Insurance Research Database, Taiwan, 2005-2018.**

| **Drug Class** | **ATC code** |
| --- | --- |
| Biguanide | A10BA02 |
| Sulfonylurea | A10BB01、A10BB07、A10BB09、A10BB12 |
| Glinide | A10BX02、A10BX03 |
| Thiazolidinedione | A10BG02、A10BG03 |
| Alpha -glucosidase inhibitors | A10BF01、A10BF02 |
| Dipeptidyl peptidase IV inhibitor | A10BH01、A10BH02、A10BH03、A10BH04、A10BH05 |
| Fixed-dose combination | A10BD02、A10BD05、A10BD07、A10BD08、A10BD09、A10BD10、A10BD11、 A10BD13 |
| Human insulin or insulin analogue | A10AB01、A10AC01、A10AB04、A10AD05、A10AB06、A10AE04、A10AE05 |
| Glucagon-like peptide-1 agonist | A10BJ01、A10BJ02 |
| Angiotensin converting enzyme inhibitor | C09AA01、C09AA02、C09AA03、C09AA04、C09AA05、C09AA06、C09AA07、C09AA08、C09AA09、C09AA16、C09BA01、C09BA02、C09BA04、C09BB04、C09BB05 |
| Angiotensin receptor blocker | C09CA01、C09CA02、C09CA03、C09CA04、C09CA06、C09CA07、C09CA08、C09CA09、C09DA01、C09DA03、C09DA04、C09DA06、C09DA07、C09DA08、C09DA09 |
| Nonsteroidal anti-inflammatory drug | M01AB01、M01AB02、M01AB03、M01AB05、M01AB06、M01AB08、M01AB11、M01AB15、M01AB16、M01AC01、M01AC02、M01AC06、M01AE01、M01AE02、M01AE03、M01AE09、M01AE04、M01AE11、M01AE16、M01AG01、M01AG02、M01AG03、M01AG04、M01AH01、M01AH02、M01AH05 |

**Table S3: Sensitivity analysis of the combined incidence of chronic kidney disease and diabetic kidney disease and related Hazard Ratios with 95% confidence intervals in participants with and without hyperglycemic crisis at diabetes diagnosis from the National Health Insurance Research Database, Taiwan, 2006-2018.**

|  | **Hyperglycemic crisis** | | | | | | | | | |
| --- | --- | --- | --- | --- | --- | --- | --- | --- | --- | --- |
| **Outcome** | **No**  **N=13242** | | **All**  **N=13242** | | **DKA**  **N=7297** | | **HHS**  **N=5185** | | **DKA-HHS**  **N=760** | |
|  | n | Rate^a^ | n | Rate^a^ | n | Rate^a^ | N | Rate^a^ | n | Rate^a^ |
| DKD or CKD^b^ | 2511 | 26.16 | 3388 | 46.60 | 1744 | 38.46 | 1426 | 60.65 | 218 | 56.64 |
| Crude HR | Ref. |  | 1.84 | (1.75–1.94) | 1.49 | (1.40-1.59) | 2.48 | (2.33-2.65) | 2.29 | (1.99-2.64) |
| Adjusted HR^c^ | Ref. |  | 1.87 | (1.77–1.97) | 1.56 | (1.46-1.65) | 2.36 | (2.21-2.52) | 2.37 | (2.06-2.73) |
| Crude SHR | Ref. |  | 1.50 | (1.43–1.58) | 1.36 | (1.28-1.44) | 1.67 | (1.56-1.78) | 1.88 | (1.63-2.16) |
| Adjusted SHR^c^ | Ref. |  | 1.53 | (1.46–1.62) | 1.41 | (1.33-1.50) | 1.66 | (1.55-1.77) | 1.93 | (1.68-2.22) |

Abbreviations: CKD, chronic kidney disease; DKA, diabetic ketoacidosis; DKD, diabetic kidney disease; HHS, hyperglycemic hyperosmolar state; HR, hazard ratio; SHR, sub-distribution hazard ratio.

^a^ Per 1,000 person-years.

^b^ Patients with outcome that occurred within 6 months after type 2 diabetes diagnosis was regarded as not having an event.

^c^ Adjusted for age, sex, socioeconomic factors, and significant comorbidities at baseline.

**Table S4: Nested case-control analysis evaluating risk factors associated with chronic kidney disease or diabetic kidney disease among participants in the hyperglycemic and non-hyperglycemic cohorts from the National Health Insurance Research Database, Taiwan, 2006-2018.**

|  | **CKD or DKD**  **N=6841** | | **Without CKD/DKD**  **N=19643** | | | **Odds ratio (95% CI)** | |
| --- | --- | --- | --- | --- | --- | --- | --- |
| **Variable** | **n** | **%** | | **N** | **%** | **Crude** | **Adjusted^a^** |
| Comorbidity^b^ |  |  | |  |  |  |  |
| HTN | 4589 | 67.1 | | 11328 | 57.7 | 1.50 (1.41–1.59) | 1.05 (0.97–1.13) |
| Heart failure | 889 | 13.0 | | 2247 | 11.4 | 1.16 (1.06–1.26) | 0.89 (0.81–0.98) |
| CAD | 1689 | 24.7 | | 4580 | 23.3 | 1.08 (1.01–1.15) | 0.89 (0.83–0.96) |
| Stroke / TIA | 1273 | 18.6 | | 3709 | 18.9 | 0.98 (0.92–1.05) | NA |
| PAD | 433 | 6.33 | | 1208 | 6.15 | 1.03 (0.92–1.16) | NA |
| Hyperlipidemia | 4193 | 61.3 | | 11018 | 56.1 | 1.24 (1.17–1.31) | 1.22 (1.15–1.30) |
| Obesity | 197 | 2.88 | | 622 | 3.17 | 0.91 (0.77–1.07) |  |
| Malignancy | 488 | 7.13 | | 2252 | 11.5 | 0.59 (0.54–0.66) | 0.53 (0.48–0.59) |
| AKI ≥1 times^c^ | 494 | 7.22 | | 898 | 4.57 | 1.63 (1.45–1.82) | 1.33 (1.18–1.50) |
| DKA ≥1 times^c^ | 2532 | 37.0 | | 5929 | 30.2 | 1.36 (1.28–1.44) | 1.56 (1.47–1.66) |
| HHS ≥1 times^c^ | 2261 | 33.1 | | 4310 | 21.9 | 1.76 (1.65–1.87) | 1.75 (1.64–1.86) |
| Treatment^d^ |  |  | |  |  |  |  |
| NSAIDs |  |  | |  |  |  |  |
| 0 days | 2560 | 37.4 | | 7112 | 36.2 | Ref. | Ref. |
| 1-90 days | 3742 | 54.7 | | 11198 | 57.0 | 0.93 (0.88–0.98) | 0.98 (0.93–1.04) |
| ≥ 91 days | 539 | 7.88 | | 1333 | 6.79 | 1.12 (1.01–1.25) | 1.09 (0.97–1.22) |
| ACEI or ARB |  |  | |  |  |  |  |
| 0 days | 3575 | 52.3 | | 13233 | 67.4 | Ref. | Ref. |
| 1-90 days | 886 | 13.0 | | 1554 | 7.91 | 2.11 (1.93–2.31) | - 1. (1.75–2.13) |
| ≥ 91 days | 2380 | 34.8 | | 4856 | 24.7 | 1.81 (1.71–1.93) | 1.69 (1.57–1.82) |

Abbreviations: ACEI, angiotensin converting enzyme inhibitor; AKI, acute kidney injury; ARB, Angiotensin receptor blocker; CAD, coronary artery disease; CI, confidence interval; CKD, chronic kidney disease; DKA, diabetic ketoacidosis; DKD, diabetic kidney disease; HTN, hypertension; HHS, hyperglycemic hyperosmolar state; NSAID, nonsteroidal anti-inflammatory drug; PAD, peripheral arterial disease; TIA, transient ischemic attack.

^a^ Adjusted for age, sex, socioeconomic factors, and significant comorbidities at baseline.

^b^ Comorbidities before the endpoint.

^c^ Events between the diagnosis of type 2 diabetes and end point.

^d^ Medication exposure within one year before the end point.
